# Rare Presentation of Hypoglycemia in a Patient with Anaplastic Large-Cell Lymphoma

**DOI:** 10.1155/2021/6843103

**Published:** 2021-12-02

**Authors:** Raed Aldahash

**Affiliations:** Department of Medicine, Ministry of National Guard Health Affairs, King Abdullah International Medical Research Center (KAIMRC), King Saud Bin Abdulaziz for Health Science, Riyadh, Saudi Arabia

## Abstract

Anaplastic large-cell lymphoma is a rare type of aggressive non-Hodgkin's lymphoma, and arriving at a final diagnosis for this tumor is a challenge for the healthcare providers. Usually, it involves the lymph nodes and extranodal tissues such as the lungs, skin, and other soft tissues. Its presentation by extending into different organs such as the liver, lungs, bones, spleen, and thyroid is rare. Thus, involvement of other organs is very rare as we found in a 54-year-old male patient, a known case of hypertension and end-stage renal disease who was on hemodialysis, who presented to the emergency department with a history of generalized weakness and weight loss of about 20 kg for two months. The tumor cells are positive for CD45, CD30, CD15, MUMi, and Ki-67 (80%) and negative for CD20, PAX-5, CD79a, CD3, CD5, CD10, BCL6, BCL2, EMA, ALK-1, and CD138. The patient was hypoglycemic and hypercalcemic and was managed accordingly. The patient was evaluated, and the third assessment showed that hypoglycemia was resolved due to dexamethasone. The patient's glucose storage was depleted most likely due to liver involvement plus poor general condition. It was asserted that the patient's hypoglycemia could be related to his underlying malignancy. Also, the patient was advised to start tablet diazoxide 45 mg three times a day (3 mg/kg/day TID) in addition to levothyroxine tablet 50 mcg once a day. Tablet diazoxide was stopped, and nutritional support was recommended. This case reveals a rare systematic ALK-1-negative anaplastic large-cell lymphoma that involves multiple organs. The main learning point from this report is that these tumors can present atypically even in adults and can be ALK-1 negative, which is contrary to the typical systematic anaplastic large-cell lymphomas that are positive for ALK.

## 1. Introduction

Lymphomas are the typical heterogeneous category of malignancies primarily of lymph nodes and are considered as a very rare type of aggressive non-Hodgkin's lymphomas [1]. More specifically, anaplastic large-cell lymphoma constitutes 5% of all cases of non-Hodgkin's lymphomas [2]. The unique property or feature of anaplastic large-cell lymphoma is the proliferation of pleomorphic cells that carry CD30, which are constantly expressed on almost all neoplastic cells [3]. This tumor is most commonly found in children and constitutes about 40% of all non-Hodgkin's lymphomas among children with a greater percentage found among males than females [2]. There is no definitive etiology of anaplastic large-cell lymphoma; however, Epstein–Barr virus has been reported to play an important role in the pathogenesis of this disease, mainly in immunocompromised patients and among cases that occur in Asian countries [4]. Two clinical types have been recognized including localized cutaneous tumors or systematic neoplasms [5]. The former category mainly constitutes CD30-positive proliferative cells including lymphoproliferative skin disorders, and these types are ALK-negative [6]. The survival rate for cutaneous neoplasms is more than 90%, while the systematic type is more aggressive that may involve the skin and other extranodal organs [7]. In contrast to these types, systematic anaplastic large-cell lymphomas are typically ALK positive [8,9]. Both types of anaplastic large-cell lymphoma, ALK positive and ALK negative, are made up of lymphoid cells having a huge cytoplasm and pleomorphic characteristics along with horseshoe-shaped nuclei [10]. It has been found that systematic anaplastic large-cell lymphomas that are ALK positive show a better prognosis than ALK-negative tumors [7]. Through the following case report, we aim to communicate our experience with healthcare providers and to discuss the management plan necessary for patients with similar presentations.

## 2. Case Report

### 2.1. Investigation

#### 2.1.1. Description of the Case

This was a 54-year-old man, a known case of hypertension and end-stage renal disease who was on hemodialysis, who presented to the emergency department with a history of generalized weakness and weight loss of about 20 kg for two months. He had been managed and treated in an external healthcare system where he was diagnosed with a case of anaplastic large-cell lymphoma. The PAN-CT revealed extensive and innumerable lesions in different organs such as the liver, lungs, bones, spleen, and thyroid. On liver biopsy, an infiltrated high-grade/undifferentiated malignant neoplasm was found. Furthermore, his serum calcium levels were very high [11], which were managed by adequate hydration and denosumab injections [12]. His family history was not remarkable.

#### 2.1.2. Clinical Findings on Examination and Laboratory Investigations at the Time of Admission

On arrival at the emergency department, the patient was found conscious, alert, oriented, looking ill, and cachexic. However, he was hemodynamically stable and on auscultation, and the chest was found to be clear with S1 and S2 being audible on auscultating the heart with no added third sound or murmur. His initial blood pressure was 101/65 mmHg, his heart rate was 65 beats per minute with an oxygen saturation of 99% on room air, and he was breathing spontaneously.  Table 1 shows the laboratory values of the patient at the time of admission.

#### 2.1.3. Assessment by the Team of the Hospital

During the hospital course, the patient was admitted as a case of tumor lysis syndrome and was evaluated by a team of specialists including specialists from hematology/oncology, nephrology, and mental health. During the hospital stay, the patient was given a reduced dose of the 1st cycle of CVP (chemotherapy protocol) due to poor performance status and very poor nutrition without significant complications. The patient was monitored during the hospital stay, and his necessary laboratory investigations were sent to assess the progress and response to treatment.

### 2.2. Diagnosis

#### 2.2.1. Results and Conclusion Based on CT Performed outside the Hospital

A right apical micronodule, measuring 5 mm, was found. There was bilateral mild-to-moderate pleural effusion with adjacent atelectasis. However, no mediastinal, hilar, or axillary lymphadenomegaly was noticed. There were diffuse osteosclerotic and osteolytic changes throughout the sternum, vertebrae, and ribs. Based on these results, a conclusion was made about the right apical parenchymal micronodule, likely metastatic within the context.

**Figure 1:**
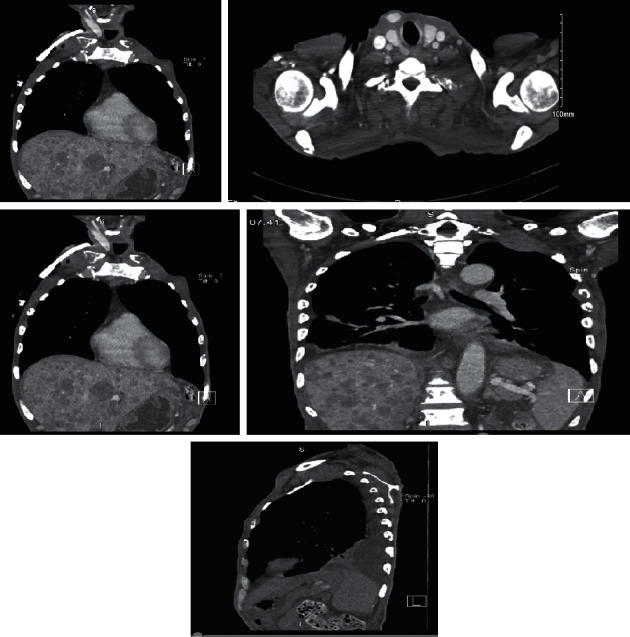
Images of the chest CT scan showing metastasis to the liver and chest wall.

Based on the findings of the CT scan, an impression of diffuse metastatic bony disease was given by the radiologist and team (Figure 1).

#### 2.2.2. Results of a Blood Smear, Biopsy, and Doppler Ultrasound

The blood smear of the patient shows normocytic normochromic red cells with mild anisocytosis. Furthermore, occasional polychromatophilic cells were seen with no significant schistocytes (less than 1%). Rare left-shifted granulocytes with mild toxic changes in neutrophils were noted. No definite circulating blasts were seen. Platelets were essentially unremarkable. Liver lesion needle core biopsy (outside materials- H 2342–20, File No. 1051463) showed anaplastic large-cell lymphoma, ALK negative. Cores of hepatic tissue were found to be infiltrated by a high-grade or undifferentiated malignant neoplasm. Poorly differentiated hepatocellular carcinoma is included in the differential diagnosis. On immunostaining, the tumor cells are positive for CD45, CD30, CD15, MUMi, and Ki-67 (80%) and negative for CD20, PAX-5, CD79a, CD3, CD5, CD10, BCL6, BCL2, EMA, ALK-1, and CD138. Findings on upper extremity venous doppler ultrasound showed patent left internal jugular, innominate, subclavian, axillary, brachial, basilic, and forearm veins. However, the left arteriovenous (AV) fistula was thrombosed. The final impression of negative study for left upper limb deep vein thrombosis (DVT) was made with thrombosed AV fistula.

#### 2.2.3. PET/CT Oncology

**Figure 2:**
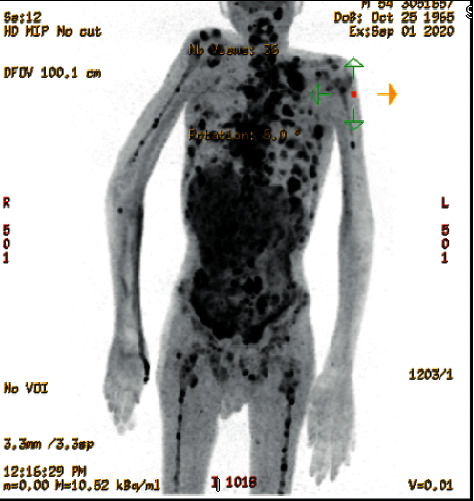
PET/CT scan of the patient admitted to the emergency department.

The study is in keeping with known lymphoma involving the skeleton extensively, liver, right posterior chest wall, left lung hilar lymph node, and possibly left upper lung lobe. Also, a suspicion of peritoneal disease was made based on the PET/CT scan (Figure 2).

### 2.3. Treatment

#### 2.3.1. Two Weeks after Admission

After two weeks of admission, the endocrinology team received a consultation regarding a hypoglycemic attack while the patient was undergoing a PET scan that was treated with 50 mL dextrose 50% in water injection with an intravenous (IV) push. The patient was seen and evaluated by the endocrinology team. After reviewing lab investigations, the patient was found to have had a low glucose profile since admission that ranges between 1.7 and 5.1 mmol/L. Hence, the patient was labeled as a case of hypoglycemia for investigations with a differential diagnosis of [1] anaplastic lymphoma with infiltration to adrenal glands, [2] end-stage renal disease (ESRD) causing hypoglycemia, [3] or nutritional inadequacy.

#### 2.3.2. Management Plan

The hospital specialists' team advised getting his serum insulin level, C-peptide, proinsulin, B-hydroxybutyrate, cortisol, and thyroid profile including TSH and free T4 at the time of hypoglycemia (blood glucose level < 2.8 mmol/L).

After taking labs, the patient was started on dextrose-infused fluids (and was watched for overload). The patient needed nutritional support and was advised to have adequate nutritional support.

### 2.4. Follow-Up and Outcomes

#### 2.4.1. Labs after Two Weeks (Second Assessment)

The patient's labs were repeated after two weeks, and the insulin was found to be < 11.50 (22.96–116.95 pmol/L), while his TSH was 21.57 (0.35–4.94 mIU/L), free T4 was 6.15 (9.00–19.00 pmol/L), IGF-1_RY was 25 (61–200 ng/mL) and 0.36 (.26–1.72 nmol/L), random glucose level was 1.7, and eGFR was 11, which further deteriorated over two weeks. However, sodium (136 mmol/L), potassium (3.1 mmol/L), chloride (102 mmol/L), BUN (18.5 mmol/L), and creatinine (243 umol/L) levels remained the same as they were at the baseline.

#### 2.4.2. Impression or Plan

It was asserted that the patient's hypoglycemia could be related to his underlying malignancy [13]. Also, the patient was advised to start tablet diazoxide 45 mg three times a day (3 mg/kg/day TID) in addition to levothyroxine tablet 50 mcg once a day. A clinical nutritional referral was made to plan proper nutritional support for him. Unfortunately, the patient was not improved on diazoxide. As planned by the hematology team, the patient was started on dexamethasone injection 40 mg IV for 5 days regarding his lymphoma situation.

#### 2.4.3. Third Assessment

The patient was evaluated, and the third assessment showed that hypoglycemia was resolved due to dexamethasone. The patient's glucose storage was depleted most likely due to liver involvement plus poor general condition. Tablet diazoxide was stopped, and nutritional support was highly recommended.

## 3. Discussion

A case of anaplastic large-cell lymphoma is presented in this case report that encroached multiple vital organs including the liver, spleen, and thyroid. The tumor cells were found to be positive for CD30, but negative for ALK-1. Both morphological and phenotypic features of systematic anaplastic large-cell lymphoma (negative for ALK-1) mimic mucosal CD30 positive T-cell lymphoproliferative disorders [14]. It is important to distinguish between ALK-positive and ALK-negative types of anaplastic large-cell lymphomas because there are differences between their clinical presentation and prognosis [15]. These tumors may be initially misdiagnosed and need a vigilant assessment plan with reasonable imaging and other investigations to diagnose correctly.

We found hypercalcemia and hypoglycemia in this case, which were associated with the tumor. The existing literature demonstrates that hypercalcemia due to neoplasms is a commonly found sign and it is the humoral hypercalcemia rather than Vit-D3-mediated hypercalcemia that is usually found in malignancies [11]. However, findings related to hypoglycemia in tumors that are the outside the pancreas, such as this anaplastic large-cell lymphoma, are rare and hardly ever reported in similar case reports. Glucose is a necessary fuel for the function of the brain, and complex neural, cellular, and hormonal controls try to maintain the glucose levels within a reasonably narrow range. Usually, we find hypoglycemia rarely in nondiabetic patients, and therefore, one may need to be ruled out for potential malignancies as we found in our case.

After evaluating the patient, we also concluded that hypoglycemia is more likely due to lymphoma and it was resolved by giving dexamethasone to the patient. The patient's glucose storage was depleted most likely due to liver involvement plus poor general condition. Although we did not obtain bone marrow biopsy or insulin-like growth factors in the blood, we presumed that this hypoglycemia is most likely due to the anaplastic large-cell lymphoma because we did not find other alternative mechanisms of severe hypoglycemia in the patient such as sepsis, adrenal insufficiency, or insulinoma [13]. Furthermore, it is likely that such hypoglycemia may also be due to overproduction of insulin-like growth factor (IGF)-2 precursor protein, which is commonly known as “big insulin-like growth factor- (IGF-) 2” [16]. This is also supported by the literature whereby large neoplasms sometimes may produce IGF-2 that can circulate in the blood followed by binding to insulin and IGF receptors. This will result in increased uptake of glucose in the tissues and reduced glucose in the blood; thereby, the liver reduces the release of glucose to the blood. This provokes the feedback mechanism and suppresses insulin, insulin-like growth factor 1, and the production of growth hormone. Thus, it is unique to find IGF-2-related lower blood glucose levels in tumors such as anaplastic large-cell lymphomas [17]. This case of a middle-aged gentleman revealed that although rare, sometimes hypoglycemia may be due to the release of IGF-2 in nonsolid neoplasms such as anaplastic large-cell lymphomas. Because most literature has shown that hypoglycemia may be associated with the uncommon tumors of the pancreas such as insulinomas, however, hypoglycemia can also be found in non-islet cell tumors outside the pancreas by following both insulin-dependent and -independent mechanisms [18].

Furthermore, the existing study by Gorden et al. who investigated 7 patients with hypoglycemia and lymphoma found low levels of insulin-like growth factor in the blood. They found these levels lower than the normal population [19]. On the other hand, some patients with hypoglycemia with Hodgkin's disease showed reduced plasma insulin levels and proinsulin. However, these patients' levels for C-peptide and insulin-like growth factor 1 were found lower, which may indicate a reasonable relationship between non-islet cell tumors such as anaplastic large-cell lymphomas and IGF-2-associated hypoglycemia.

To recapitulate, it may be easy to pay attention to hypoglycemia in diabetic patients or patients diagnosed with islet cell tumors. However, it is important to pay attention to the history of the patient, histological features, and other investigations that may help to reach the cause of severe hypoglycemia in patients not suspected of islet cell tumors. A holistic approach should be made to make a connection between hypoglycemia non-islet cell tumors such as anaplastic large-cell lymphomas to avoid missing such cases. One may tend to miss these tumors when a patient presents with hypoglycemia because it is not a common finding in such patients with nonsolid tumors who may be either missed or diagnosed late with anaplastic large-cell lymphomas. Therefore, keeping hypoglycemia in mind as a possible finding in such patients, such patients presenting with severe hypoglycemia should be ruled out for nonsolid tumors or hematological diseases to manage them on time and save their lives. For example, one may plan to conduct an initial evaluation to determine the cause of hypoglycemia by taking a detailed history to find out the nature of symptoms and how these are related to time, for example, is it related to a meal or not [20]. Furthermore, the physician should also evaluate the patient for any signs and symptoms of the tumor as well as medications taken by the patient. This can be followed by laboratory work-up of insulin-like growth factors, insulin, C-peptide, and plasma glucose.

### 3.1. Learning Points

Hypoglycemia is a rare finding among patients diagnosed with anaplastic large-cell lymphomas. One should suspect hypoglycemia in patients diagnosed with nonsolid tumors such as anaplastic large-cell lymphomas. Patients presenting with severe hypoglycemia should be ruled out for nonsolid tumors or hematological disease.

## Figures and Tables

**Table 1:** Clinical findings on admission to the Emergency Department (ED) of the hospital.

Assessment on arrival by ED staff	Normal value	Clinical findings and labs of the patient
Blood pressure, mmHg	120/80	101/65
Heart rate (beats per minute)	60–100	65
Oxygen saturation, (Spo2), %	≥ 95	99
Sodium, mmol/L	136–145	136
Potassium, millimoles per liter (mmol/L)	3.5–5.1	3.1
Serum chloride, milliequivalents per liter (mEq/L)	98–107	102
Random glucose	2.9–7.8	5.6
Blood urea nitrogen (BUN), mg/dL	3.0–9.2	18.5
Creatinine, umol/L	64–110	243
CO2, mmol/L	22–29	17
AGAP, mmol/L	7–15	20
Calcium, mmol/L	2.10–2.5	1.88, 1.74
eGFR	>= 60 mL/min/1.73 m^2^	26
Phosphorus, mmol/L	0.74–1.52	0.97
Magnesium, mmol/L	0.66–1.07	0.74
Uric acid, umol/L	210–420	153
Adj Ca, mmol/L	2.10–2.55	2.06
Hgb, gm/L	135–180	90
Hct, L/L	0.420–0.540	0.273
MCV, fL	76.0–96.0	89.2
MCH, pg	27.0–32.0	29.3
Platelet	150–400 x 10^9^/L	98
MPV, fL	7.4–10.4	5.9
PT, second (s)	9.38–12.34	12.2
INR	0.80–1.20	1.1
PT, second (s)	24.84–32.96	26.5
GGT	12–64 U/L	505
LDH	125–220 U/L	2944
Alk Phos	40–150 U/L	332
Bili T	3.4–20.5 umol/L	8.3
AST	5–34 U/L	31
ALT	5–55 U/L	21
Total protein	60–83 g/L	55

## Data Availability

The patient's investigations and imaging scans are available upon request.
